# The Effect of Heat Stress on Sensory Properties of Fresh Oysters: A Comprehensive Study Using E-Nose, E-Tongue, Sensory Evaluation, HS–SPME–GC–MS, LC–MS, and Transcriptomics

**DOI:** 10.3390/foods13132004

**Published:** 2024-06-25

**Authors:** Bing Fu, Chang Fang, Zhongzhi Li, Zeqian Zeng, Yinglin He, Shijun Chen, Huirong Yang

**Affiliations:** 1 College of Marine Sciences, South China Agricultural University, Guangzhou 510640, China; 2 Zhongshan Innovation Center of South China Agricultural University, Zhongshan 528400, China

**Keywords:** high temperature, seafood, sensory evaluation, metabolism

## Abstract

Heat stress has received growing concerns regarding the impact on seafood quality. However, the effects of heat stress on the sensory properties of seafood remain unknown. In this study, the sensory properties of fresh oyster (*Crassostrea ariakensis*) treated with chronic heat stress (30 °C) for 8 weeks were characterized using electronic nose, electronic tongue, sensory evaluation, HS–SPME–GC–MS, LC–MS and transcriptomics. Overall, chronic heat stress reduced the overall sensory properties of oysters. The metabolic network constructed. based on enrichment results of 423 differential metabolites and 166 differentially expressed genes, showed that the negative effects of chronic heat stress on the sensory properties of oysters were related to oxidative stress, protein degradation, lipid oxidation, and nucleotide metabolism. The results of the study provide valuable insights into the effects of heat stress on the sensory properties of oysters, which are important for ensuring a sustainable supply of high-quality seafood and maintaining food safety.

## 1. Introduction

Seafood, as a primary source of protein for humans, accounts for 17% of the total consumption of animal protein worldwide [1]. However, with the intensification of global warming, heat stress has had increasingly negative impacts on the physiology and survival of marine organisms, posing a growing threat to seafood production [2]. Additionally, abnormal metabolism in marine organisms induced by heat stress may adversely affect seafood quality. Given the continuous growth of the global population and the increasing reliance on seafood [1,2,3], assessing the impact of heat stress on seafood quality is crucial for ensuring sustainable seafood supply and guaranteeing food security.

Seafood outshines other meat sources due to its rich nutritional profile, encompassing protein, fatty acids, and minerals, which contribute importantly to human health [2,3]. Heat stress has been reported to induce excessive production of reactive oxygen species (ROS). Excess ROS accelerate the oxidation of biological components, such as lipids, proteins, and nucleic acids, and the oxidation process may produce toxic oxidation products that may be harmful to human health [4]. Generally, heat stress (HS) is classified into two categories based on duration: acute HS (e.g., hours) and chronic HS (e.g., days or weeks) [4,5]. However, it is a concern that the nutritional quality of seafood is more vulnerable to chronic heat stress rather than acute heat stress [2,6]. This effects were mainly reflected in a decrease in unsaturated fat content, and protein, and an increase in saturated fat [2]. In addition to the nutritional properties of seafood, sensory properties should also be emphasized, as sensory properties contribute to consumers’ choices in seafood consumption. Recently, several studies have shown that chronic heat stress disrupted the metabolic homeostasis of marine organisms served as seafood [5,7]. Therefore, the sensory properties based on metabolites may also be altered by heat stress. However, the current research on the effect of chronic heat stress on the sensory properties of seafood is still limited.

Sensory properties, like flavor and taste, are highly associated with flavor precursors, which can be classified into water-soluble and lipid-soluble metabolites. Water-soluble precursors, including amino acids, nucleotides, and vitamins, are particularly important. Amino acids, such as glutamic acid (Glu), serine (Ser), and glycine (Gly), have a crucial role in influencing flavor and taste [8]. In particular, 5′-nucleotides are closely related to freshness and umami taste [9]. Lipid-soluble precursors are mainly lipid metabolites, including unsaturated fatty acids that, when oxidized, generate volatile flavor compounds, such as aldehydes, ketones, and alcohols, thereby influencing the overall flavor [10]. Furthermore, these metabolites interact synergistically to impact the overall sensory properties of seafood.

Currently, research on the effects of heat stress on oysters has focused on growth and development, disease, and nutrient characterization, but less on the sensory characteristics of oysters. Given that the sensory properties of oysters determine consumer purchasing choices, it is imperative that effects of heat stress on the sensory properties of oysters are investigated. The oyster *Crassostrea ariakensis*, commonly found throughout South China, is known for its rapid growth and tolerance to high salinity. Due to its nutritional value and freshness, it is very popular among consumers. In 2022, the annual production of oysters in Guangxi, China, is 672,000 tons, ranking first in China [11]. As an estuarine species, this oyster is regarded as one of the most heat-sensitive species [12]. Therefore, in this study, the sensory properties of fresh oyster *C. ariakensis* treated with chronic heat stress (30 °C) for 8 weeks were characterized, applying electronic nose, electronic tongue, sensory evaluation, headspace solid-phase microextraction gas chromatography–mass spectrometry (HS–SPME–GC–MS), liquid chromatography–mass spectrometry (LC–MS) and transcriptomics. The aims of the study were to (i) explore the effects of heat stress on the sensory properties of oysters, (ii) identify key metabolites involved in heat stress affecting the sensory properties of oysters, and (iii) explore potential metabolic mechanisms on sensory characteristics changes of oysters under heat stress.

## 2. Materials and Methods

### 2.1. Heat Stress Treatment and Sample Collection

Oysters from the ZhenHai Bay were collected for the experiments. Healthy and adult oysters without barnacles were selected and epiphytes were removed from the shell surface. The oysters were cultured using artificial seawater conditions at a temperature of 20.10 ± 0.01 °C, pH 8.06 ± 0.04, salinity 20.1 ± 0.03 g·L^−1^ and dissolved oxygen (DO) 6.2 ± 0.12 mg·L^−1^. They were fed twice daily with *Isochrysis galbana* and acclimated under laboratory conditions for one week prior to the start of the experiments.

Oysters were divided into two groups: a heat stress group (HS, 30 °C) and a normal group (NT, 20 °C). The temperature of 30 °C is commonly used in heat stress treatments [13,14], and corresponds to the predicted temperature of warming seawater at the end of the century [15]. Therefore, 30 °C was used as the exposure temperature for the heat stress group, while 20 °C represented the annual mean seawater temperature. Each treatment consisted of three biological replicates, conducted in three independent tanks (150 L) with 30 individuals per tank. Normal and high temperatures were achieved and maintained using portable aquarium water chillers (GeekTeches STC-200, Shanghai Sishun Co. Ltd., Shanghai, China) and thermostatically monitored electric heaters (AR-4 J, SUNSUN, Zhejiang, China), respectively. In the heat stress group, the water temperature was gradually raised from approximately 20 °C to 30 °C over a period of ten days (one degree per day) to prevent thermal shock in the oysters prior to the formal experiment. After starting the experiment, the oysters were fed twice daily with *Isochrysis galbana* (08:00 and 16:00). The salinity (20.1 ± 0.05 g·L^−1^) and pH (8.06 ± 0.03) of the water in each tank remained stable during the entire experiment. After 8 weeks, three oysters were randomly selected from each treatment and dissected. The soft tissues, excluding the visceral mass, were collected in centrifuge tubes with caps, quickly frozen, and stored at −80 °C. All the experiments were performed following the animal ethics guidelines approved by the Ethics Committee of South China Agricultural University (2023G031).

### 2.2. Analysis of Electronic Nose, Electronic Tongue, Sensory Evaluation and Proximate Composition

Odor characteristics of oysters were assessed using a PEN3 Portable Electronic Nose (E-Nose) (Insent Company, Atsugi, Japan), following a previously established method [16]. Specifically, 5 g of sample was homogenized with 2 mL of 0.18 g/mL NaCl solution. The mixture in the 5-mL E-nose sampling bottle was incubated at 60 °C for 10 min. Clean dry air was used as the carrier gas at a flow rate of 150 L/min. The measurement phase was of 120 s duration. 

The bitterness, umami, saltiness, richness, sourness, and astringency of oysters were determined using an electronic tongue (E-tongue) (TS-5000Z, Intelligent Sensor Technology, Inc., Atsugi, Japan), following a previously established method [17]. Specifically, 2.0 g of sample was mixed with a certain volume of distilled water to extract taste substances. Having initially washed the sensors in a cleaning solution (90 s) and reference solutions (120 s + 120 s), they were dipped in the sample solutions for 30 s. 

Sensory evaluation is one of the most important methods to evaluate the quality of aquatic products. The sensory panel consisted of ten panelists that were all selected, tested, and trained in descriptive analysis according to ISO 11035-1994 [18]. Training of panelists included three separate sessions. The 1st session was dedicated to the generation of vocabulary. Panelists received oyster samples and were asked to identify tastes, smells, and appearance of oysters. A panel discussion followed to reach consensus regarding the type and number of attributes. The 2nd session included evaluation of oyster samples in order to choose the most representative attributes among those generated during the 1st session [19]. The 3rd session was dedicated to the clarification of some attributes the panelists considered challenging. The final list of attributes, divided in sensory modalities, along with their definition can be found in Table S1. Specifically, after the *C. ariakensis* were steamed for 3 min, they were presented to panelists in the form of a half shell for evaluation. To reduce error, oysters per treatment were given three random numbers, respectively. Sensory evaluators’ perception intensity of specific sensory attributes, from weak to strong, was represented by a corresponding score from 1 to 10, and the final score of sensory evaluation was the average value.

The moisture, protein, ash, and lipid contents of oyster samples were determined according to the method described by previous research [5,20]. The samples were dried at 105 °C overnight in a hot air oven until constant weight was obtained for the determination of moisture content. The crude protein content was analyzed by the semi-automated Kjeldahl system. Ash content was analyzed by incinerating the dried oyster samples in the muffle furnace at 550 °C overnight. The crude lipid content was determined using the Soxhlet extraction method. 

### 2.3. Metabolite Analysis by LC–MS

Three samples from each group were used to analyze for LC–MS. Specifically, a 50 mg sample was added to a 2 mL centrifuge tube and a 6 mm diameter grinding bead was added. 400 μL of extraction solution (methanol/water = 4:1 (*v*/*v*)) containing 0.02 mg/mL of internal standard (L-2-chlorophenylalanine) was used for metabolite extraction. Samples were ground by the Wonbio-96c (Shanghai Wanbo Biotechnology Co., Ltd., Shanghai, China) frozen tissue grinder for 6 min (−10 °C, 50 Hz), followed by low-temperature ultrasonic extraction for 30 min (5 °C, 40 kHz). The samples were left at −20 °C for 30 min, centrifuged for 15 min (4 °C, 13,000× *g*), and the supernatant was transferred to the injection vial for LC–MS/MS analysis.

The LC–MS/MS analysis of sample was conducted on a Thermo UHPLC-Q Exactive HF-X system equipped with an ACQUITY HSS T3 column (100 mm × 2.1 mm i.d., 1.8 μm; Waters, Milford, MA, USA) at Majorbio Bio-Pharm Technology Co., Ltd. (Shanghai, China). The mobile phases consisted of 0.1% formic acid in water/acetonitrile (95:5, *v*/*v*) (solvent A) and 0.1% formic acid in acetonitrile/isopropanol: water (47.5:47.5, *v*/*v*) (solvent B). Positive ion mode separation gradient: 0–3 min, mobile phase B was increased from 0% to 20%; 3–4.5 min, mobile phase B was increased from 20% to 35%; 4.5–5 min, mobile phase B was increased from 35% to 100%; 5–6.3 min, mobile phase B was maintained at 100%; 6.3–6.4 min, mobile phase B was decreased from 100% to 0%; 6.4–8 min, mobile phase B was maintained at 0%. Separation gradient in negative ion mode: 0–1.5 min, mobile phase B rises from 0 to 5%; 1.5–2 min, mobile phase B rises from 5% to 10%; 2–4.5 min, mobile phase B rises from 10% to 30%; 4.5–5 min, mobile phase B rises from 30% to 100%; 5–6.3 min, mobile phase B linearly maintains 100%; 6.3–6.4 min, the mobile phase B decreased from 100% to 0%; 6.4–8 min, the mobile phase B was linearly maintained at 0%. The flow rate was 0.40 mL/min and the column temperature was 40 °C.

The pretreatment of LC/MS raw data was performed by Progenesis QI (Waters Corporation, Milford, MA, USA) software (version 3.0), and a three-dimensional data matrix in CSV format was exported. The information in this three-dimensional matrix included sample information, metabolite name and mass spectral response intensity. Internal standard peaks, as well as any known false positive peaks (including noise, column bleed, and derivatized reagent peaks), were removed from the data matrix, made de-redundant and peak pooled. At the same time, the metabolites were identified by searching database, and the main databases were the HMDB (http://www.hmdb.ca/, accessed on 25 January 2024) and Metlin (https://metlin.scripps.edu/, accessed on 25 January 2024). To identify differential metabolites (DMs), the detected metabolites were subjected to orthogonal partial least squares discriminant analysis (OPLS–DA). Metabolites with a variable importance of projection (VIP) value > 1, a significance level of *p* < 0.05 and |log_2_(fold change)| ≥ 0.26 were considered statistically significant. The metabolic pathways of the DMs were analyzed and enriched using the Kyoto Encyclopedia of Genes and Genomes (KEGG) (https://www.metaboanalyst.ca/, accessed on 25 January 2024).

### 2.4. Volatile Metabolite Analysis by HS–SPME–GC–MS

The volatile components from oyster tissue samples were collected applying solid phase microextraction (SPME) and measured using a Shimadzu-QP2010 GC–MS system (Shimadzu, Kyoto, Japan). The processing methods for the oyster tissue samples were based on a previous study [16]. The chromatographic responses (peak area counts) of the volatile compounds were recorded, and the relative content of the volatile components was determined by normalizing the peak areas.

### 2.5. Library Construction and RNA–Seq Analysis

Total RNA was extracted from oyster sample using the TRIzol method, followed by RNA purification, reverse transcription, library construction, and sequencing at Shanghai Majorbio Bio-pharm Biotechnology Co., Ltd. (Shanghai, China) as per the manufacturer’s instructions (Illumina, San Diego, CA, USA). The RNA–seq transcriptome library was prepared using Illumina^®^ Stranded mRNA Prep, Ligation kit with 1 μg of total RNA. Subsequently, the raw paired-end reads were trimmed and quality controlled using fastp software (version 0.20.0) [21] with default parameters. Then clean reads were separately aligned to oyster reference genome (CNA0022698, https://ftp.cngb.org/pub/CNSA/data3/CNP0001149/CNS0353631/CNA0022698/, accessed on 4 January 2024) with orientation mode using HISAT software (version 2.0.4). The mapped reads of each sample were assembled by StringTie in a reference-based approach.

To identify differential expression genes (DEGs) between two groups, the expression level of each transcript was quantified using the transcripts per million reads (TPM) method. Differentially expressed gene (DEG) identification was conducted with DESeq. DEGs were identified as genes with |log_2_(fold change)| > 2 and *p* value < 0.05. Gene Ontology (GO) of Genes and Kyoto Encyclopedia of Genes and Genomes (KEGG) databases were applied to identify the function profile. 

### 2.6. DHE Staining and Analysis of Antioxidant Indexes

To detect superoxide radicals in tissues, dihydroethidium (DHE) staining was conducted following a previous study [5]. Oyster samples were frozen in Tissue-Tek OCT embedding medium (Tissue-Tek 4583, Tokyo, Japan) and sectioned into 5-um-thick slices. The resulting sections were stained red using DHE solution (Sigma, D7008, USA). Observation and photography were performed using an Olympus BX53 light microscope, and the resulting images were quantified using Image J software (version 1.8.0) (https://imagej.nih.gov/ij/download.html, accessed on 28 January 2024).

Commercial kits (NanJing JianCheng Bioengineering Institute, Nanjing, China) were employed to measure malondialdehyde (MDA) (No. A003-1), superoxide dismutase (SOD) (No. A001-1-2), glutathione (GSH) (No. A006-1-1), and catalase (CAT) (No. A007-1-1) content in the oyster tissues, following the manufacturer’s instructions.

### 2.7. Statistical Analysis

To analyze the differences between the HS (heat stress) and NT (normal temperature) groups in the E-nose and E-tongue responses, radar graph and T-test were applied. The correlations between E-nose and E-tongue responses and DMs were evaluated through Procrustes analysis, Two-Way Orthogonal Partial Least Squares (O2PLS), and Pearson’s analysis. The correlations between DMs and DEGs were analyzed using a nine-quadrant diagram. In order to analyze the sensory properties for regulatory network, DEG data were firstly imported into the string database (https://string-db.org/, accessed on 28 January 2024) to establish the protein–protein interaction (PPI), and the hug genes were analyzed by the CytoHubba in Cytoscape (v. 3.8.2). Unless specified otherwise, all data were reported as the means ± standard deviation (SD). Statistical significance was determined using a significance level of *p* < 0.05 for all tests conducted.

## 3. Results and Discussion

### 3.1. The Effect of Chronic Heat Stress on Sensory Properties and Proximate Composition of Oyster C. ariakensis

**Figure 1:**
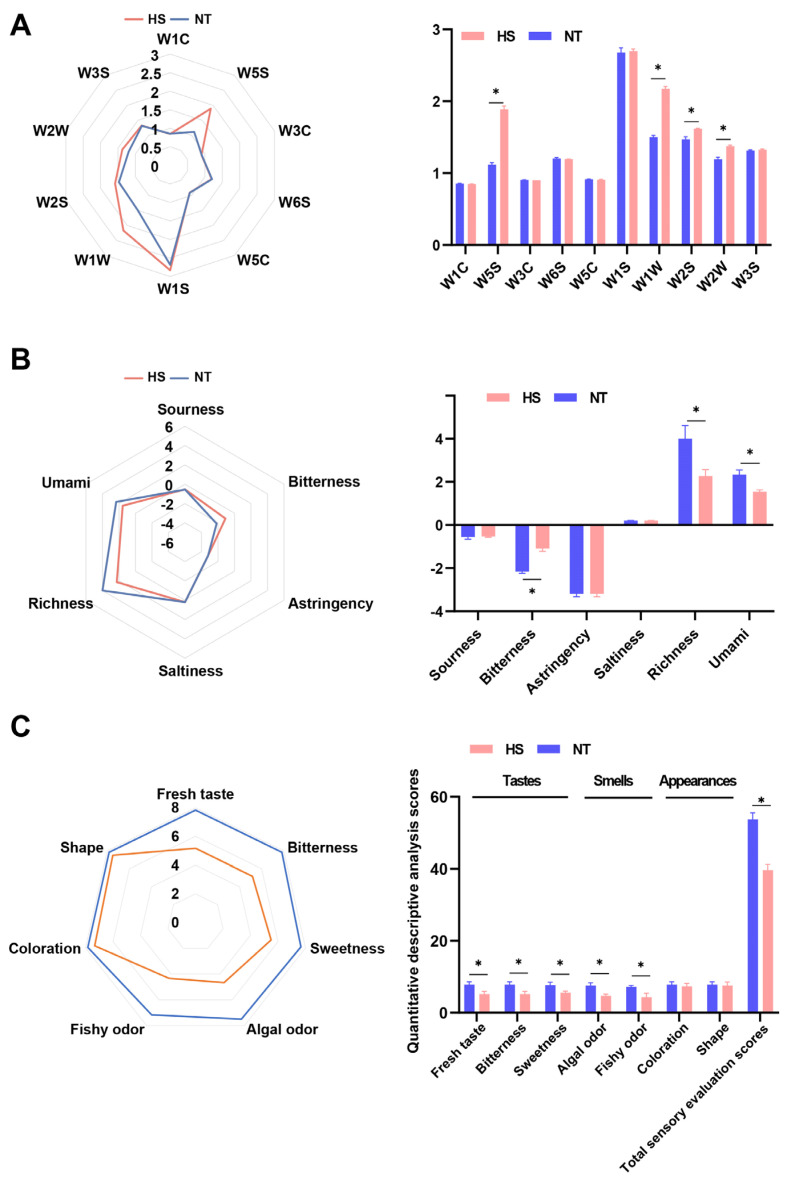
The effect of chronic heat stress on sensory properties of oyster *C. ariakensis*: (**A**) Radar and bar chart for electronic nose responses; (**B**) Radar and bar chart for electronic tongue responses; (**C**) Radar and bar chart for quantitative descriptive analysis. The total scores for sensory evaluation are the sum of the sensory scores for tastes (fresh taste, bitterness, and sweetness), smells (algal odor and fishy odor) and appearance (coloration and shape). Higher sensory evaluation scores for tastes, smells, and appearance indicate greater acceptability. NT, normal group. HS, heat stress group. Statistical analyses were performed using Student’s *t*-test, * *p* < 0.05.

The electronic nose (E-nose) serves as a valuable tool for analyzing the smell of meat [22]. The response of the E-nose to oyster *C. ariakensis* treated with heat stress is shown in Figure 1A. Compared to the NT group, strong responses were observed from the W1W (sensitive to sulfides and pyrazines), W2W (organic sulfides), W5S (nitrogen oxides), and W2S (alcohols, aldehydes, and ketones) receptors in the HS group (*p* < 0.05). Responses from W3S (sensitive to long-chain alkanes), W6S (hydrides), W1C (aromatic components, benzene), W3C (aroma, ammonia), W1S (methyl groups) and W5C (alkenes, short-chain aromatic compounds) were not significant between the NT group and the HS group (*p* > 0.05). These findings indicated that heat stress may influence the volatile contents of oyster *C. ariakensis*, thereby altering its smell. The use of the electronic tongue (E-tongue) can objectively can analyze the taste characteristics of meat [23]. As shown in Figure 1B, compared to the NT group, the HS group showed lower levels of umami and richness, and higher levels of bitterness (*p* < 0.05). However, no significant differences were observed in terms of saltiness, astringency, and sourness between the two groups (*p* > 0.05). The results suggested that heat stress reduces the taste of oyster *C. ariakensis*. In addition, the results of the sensory evaluation revealed lower taste, smell, and total sensory evaluation scores in the HS group than in the NT group (Figure 1C). Therefore, the results showed that heat stress reduced the taste and changed the smell of oyster *C. ariakensis*.

**Figure 2:**
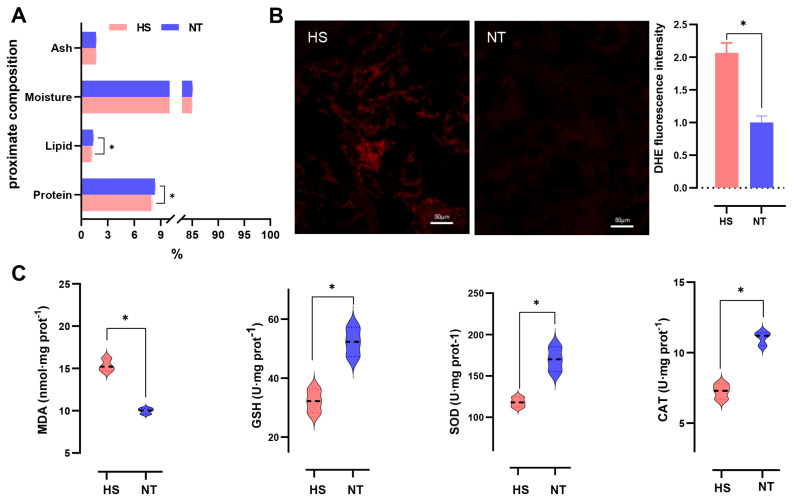
The effect of chronic heat stress on proximate composition and antioxidant abilities of oyster *C. ariakensis*: (**A**) Proximate composition; (**B**) ROS was revealed by DHE staining; (**C**) analysis of antioxidant abilities (GSH, SOD, CAT and MDA); NT, normal group. HS, heat stress group. Statistical analyses were performed using Student’s *t*-test, * *p* < 0.05.

The proximate composition of food, including moisture, ash, protein, and lipid, serves as a measure of nutritional quality. Seafood, compared to other meats, is considered highly nutritious and essential for human health and well-being [2]. As shown in Figure 2A, chronic heat stress led to a significant reduction in protein and lipid content (*p* < 0.05). Interestingly, reductions in protein and lipid had also been observed in other aquatic products (e.g., *Oreochromis niloticus* [5], *Dicathais orbita* [24]) under heat stress. Several studies have shown that heat stress induces an imbalance between reactive oxygen species (ROS) production and the action of the antioxidant defense system, which in turn leads to the accumulation of excess ROS in the cell. Excess ROS cause a dramatic increase in oxidative damage to cells, which can lead to damage to protein and lipid structures [5,25]. In our study, there was a significant increase in ROS levels (Figure 2B), a decrease in antioxidant enzyme activity (GSH, glutathione; CAT, catalase; SOD, superoxide dismutase) and an increase in MDA (malondialdehyde, a lipid peroxidation product) in the HS group compared to the NT group (Figure 2C), suggesting that the decrease in protein and lipid may be related to the increase in ROS levels and the decrease in antioxidant abilities induced by heat stress. In short, these results highlighted the detrimental impacts of heat stress on the nutritional attributes of oyster *C. ariakensis*. 

### 3.2. The Effect of Chronic Heat Stress on Non-Volatile Metabolites of Oyster C. ariakensis

**Figure 3:**
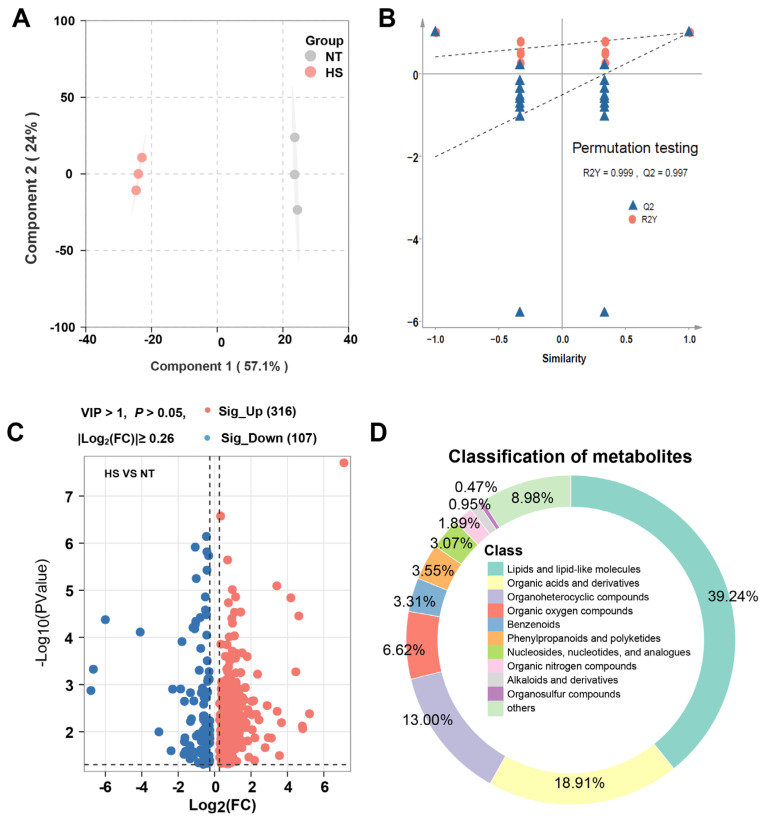
The effect of chronic heat stress on non-volatile metabolites of oyster *C. ariakensis*: (**A**) OPLS–DA; (**B**) OPLS–DA model permutation test plots; (**C**) Volcano plot; (**D**) Categorical pie chart of differential metabolites. NT, normal group. HS, heat stress group.

LC–MS is a highly effective method for qualitative analysis in taste analysis, particularly for the analysis of flavor precursor substances and non-volatile components, such as lipids, amino acids, and nucleic acids [26]. After conducting an initial analysis of metabolites in the samples from the HS and NT groups using LC–MS, we employed OPLS–DA to explore and examine the differences between these two groups (Figure 3A). The plot generated from the OPLS–DA model demonstrated a distinct separation between the HS and NT groups, indicating that heat stress-induced significant alterations in the metabolic profiles of oyster *C. ariakensis*. The OPLS–DA model yielded R^2^Y, and Q^2^ values of 0.999 and 0.997 (Figure 3B), respectively, demonstrating the high suitability of the model for analysis. The differential metabolites (DMs) were identified using VIP scores obtained from the OPLS–DA model (VIP > 1). A volcano plot revealed 423 DMs that met the threshold criteria of VIP > 1.0, *p* < 0.05, and |log_2_(FC)| ≥ 0.26. Out of these, 316 metabolites were up-regulated, while 107 were down-regulated (Figure 3C and Table S2). The DMs were identified and categorized through database (HMDB and Metlin) searches. As shown in Figure 3D, DMs were categorized into 10 groups. DMs were mainly composed of compounds such as lipids and lipid-like molecules (39.24%), organic acids and their derivatives (18.91%) and nucleosides, nucleotides, and analogues (3.07%), which implies that heat stress may produce the modulation of these differential metabolites to affect the smell and taste of oysters.

**Figure 4:**
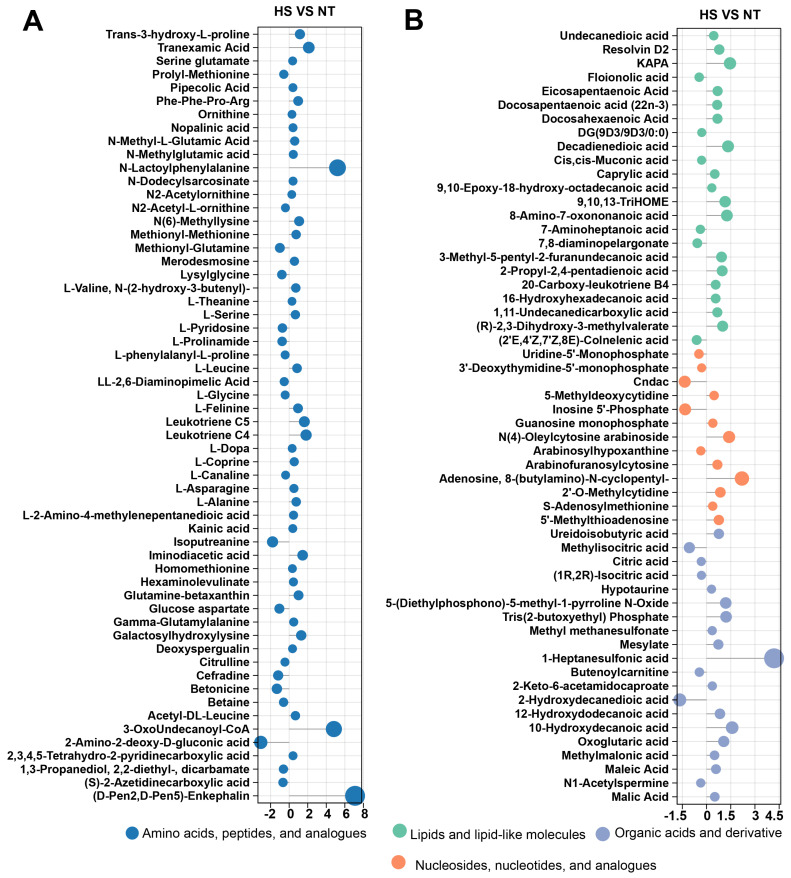
The expression profiles of amino acids, fatty acids, nucleotides, and organic acid-related precursors: (**A**) Lollipop graph for fatty acids, nucleosides, organic acids; (**B**) Lollipop graph for amino acids. The precursor substances meet the criteria: VIP > 1.0, *p* < 0.05, and |log_2_(FC)| ≥ 0.26. NT, normal group. HS, heat stress group.

Sensory properties, like flavor and taste, are highly associated with precursor substances, which are classified into water-soluble and lipid-soluble metabolites. These precursor substances mainly include amino acids, fatty acids, nucleotides, organic acids, etc. Based on the results of LC–MS analysis, lollipop graphs were used to display the expression profiles of these precursor substances. Sixty compounds related to amino acids (Figure 4A), twenty-three compounds related to fatty acids, twenty compounds related to organic acids, and thirteen compounds related to nucleotides (Figure 4B) were identified. 

Free amino acids, derived from protein degradation, serve as crucial flavor compounds and precursors to important volatile flavor compounds. In seafood like oysters, amino acids play a pivotal role as key taste-active components, contributing significantly to product quality [8]. In our study, the sweet amino acid glycine (Gly) was down-regulated, while the bitter amino acid leucine (Leu)was up-regulated (Figure 4A), indicating heat stress reduced the taste of oyster *C. ariakensis*. In addition, peptides derived from protein degradation, are also important taste compounds and flavor precursors. The characteristics of peptides are determined by various factors, including peptide chain length, amino acid composition, sequence, and spatial structure. Small molecule peptides predominantly rely on the inherent taste of their constituent amino acids. Peptides containing hydrophilic amino acids, like glutamic acid (Glu) or asparaginic acid (Asp), at the end of the side chain tend to exhibit umami taste, while peptides with hydrophobic amino acids, such as arginine (Arg), leucine (Leu), proline (Pro), phenylalanine (Phe), etc., at the end of the side chain typically exhibit bitter taste [26]. In our results, compared to NT, one up-regulated bitter peptide (Phe-Phe-Pro-Arg) was found in HS group (Figure 2A), indicating that heat stress increased bitter peptides and reduced umami flavor in oyster *C. ariakensis*.

Lipids not only play a role in providing nutrition, but also in meat flavor and taste formation [16,27]. In the present study, it was observed that heat stress decreased the lipid content of oyster *C. ariakensis* (Figure 2B). The results of LC–MS analysis showed that saturated fatty acids (including undecane-dioic acid, caprylic acid, decadiene-dioic acid, etc.) were up-regulated, while unsaturated fatty acids (including cis-Muconic acid and (2′E,4′Z,7′Z,8E)-Colnelenic acid) were down-regulated in HS relative to NT (Figure 4B). Previous studies have shown that heat stress triggers strong oxidative stress not only in terrestrial organisms [28,29], but also in aquatic organisms [5,7]. Under oxidative stress, lipids, especially unsaturated fatty acids, are readily oxidized to produce compounds such as alcohols, aldehydes, and ketones. Therefore, the decrease in polyunsaturated fatty acids in oyster *C. ariakensis* may be due to oxidation. 

5′-Nucleotides are closely related to the taste of seafood [9]. When inosine monophosphate acid (IMP) is present alone, it does not enhance flavor significantly. However, when it synergistically interacts with sodium glutamate, it significantly intensifies the overall flavor profile [30]. In our study, heat stress reduced the IMP content in oysters. Notably, the main pathway for the degradation of adenosine 5′-triphosphate (ATP) in oysters involves consecutive steps: ATP → ADP → AMP → IMP → HxR → Hx, with hypoxanthine (Hx) resulting in a bitter taste [31]. The LC–MS results demonstrated that heat stress caused an increase in the content of the bitter substance hypoxanthine (Figure 4B), which was consistent with the results of E-tongue analysis. In addition, several studies have shown that heat stress induced metabolic abnormalities and increases the consumption of ATP [13,14], which was provided by the breakdown of lipids, glucose, and proteins and metabolized by the TCA cycle. ADP generated from ATP hydrolysis is metabolized to produce bitter Hx, which explains why heat stress caused a decrease in IMP as well as an increase in bitter Hx in oyster *C. ariakensis*. These findings suggested that heat stress may induce the degradation of IMP and increase the levels of hypoxanthine, consequently affecting the flavor of oyster *C. ariakensis*.

Organic acids are crucial contributors to the intricate and distinctive taste profiles of oysters. The results of LC–MS showed that several types of organic acids were altered in oyster *C. ariakensis* under heat stress. Figure 4A,B showed a decrease in citric acid and betaine, as well as an increase in malic acid in the HS group compared to the NT group. Citric acid, with its mild and crisp acidic flavor, contributes to the overall taste sensation of oyster [32]. Malic acid is characterized by its slightly fruity and sour taste profile [8]. Betaine, as a primary alkaloid compound, imparts a distinct sweetness and contributes to the overall flavor profile of seafood [33]. Our results indicated that heat stress altered the above organic acids, potentially reducing the taste of oyster *C. ariakensis*.

### 3.3. The Effect of Chronic Heat Stress on Volatile Compounds of Oyster C. ariakensis

**Figure 5:**
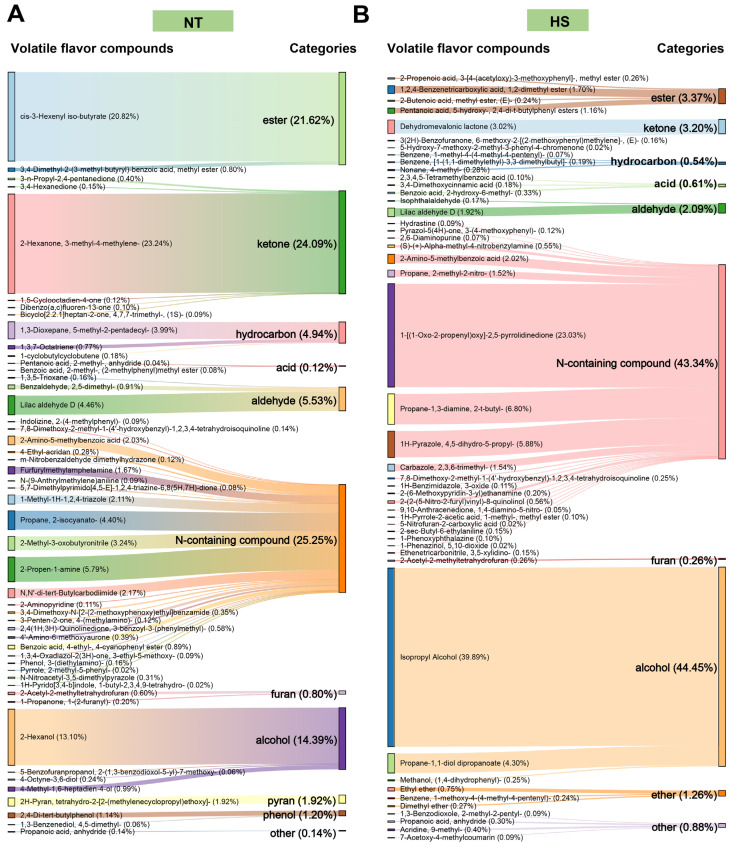
The effect of chronic heat stress on volatile compounds of oyster C. *ariakensis*: (**A**) Relative content (%) of volatile compounds for the NT group; (**B**) Relative content (%) of volatile compounds for the HS group. NT, normal group. HS, heat stress group.

To evaluate the impacts of heat stress on the volatile compounds of oyster *C. ariakensis*. HS–SPME–GC–MS was utilized to analyze the relative content and number of volatile compounds in the HS and NT groups. A total of 92 volatile compounds were identified. Figure 5A,B illustrate significant differences in the numbers and relative content of volatile compounds between the HS and NT groups (Table S3). Among these, 47 volatiles were identified in the HS (four esters, three ketones, three hydrocarbons, three acids, two aldehydes, twenty-one N-containing compounds, one furan, three alcohols, three ethers, and four other compounds). In the NT group, 50 volatiles were discovered including two esters, six ketones, three hydrocarbons, two acids, three aldehydes, twenty-four N-containing compounds, two furans, four alcohols, one pyrans, two phenols, and one other compound. In the HS group, the highest relative content (44.45%) was observed for alcohols, followed by N-containing compounds (43.34%). Conversely, in the NT group, N-containing compounds were identified as the most prominent volatiles, with a relative content of 25.25%, followed by ketones (24.09%). Notably, ethers were only detected in the HS group, while pyrans and phenols were exclusively identified in the NT group. The observed findings strongly suggested that heat stress exerted significant impacts on the composition of volatile compounds in oyster *C. ariakensis*.

Alcohols, derived from the reaction of lipoxygenase in polyunsaturated fatty acids, are a group of volatile compounds that play a crucial role in influencing flavor profiles [34]. The volatile alcohol content in the HS group (44.45%) was higher than that in the NT group (14.39%). Eight alcohols were identified in the HS group, while only three were found in the NT group. Among them, isopropyl alcohol, which imparts alcohol, musty and woody flavors [35], was the predominant alcohol in the HS group, whereas 2-hexanol, with green and sharp flavors [36], was the main alcohol in the NT group. 

Aldehydes, produced predominantly through lipid oxidation and degradation reactions, contribute significantly to the flavor profile due to their low odor threshold [37]. Compared to NT (5.53%), the aldehyde content in HS (2.09%) was lower. Three aldehydes were observed in NT, while only two were found in HS. Among them, lilac aldehyde dominated the aldehyde compounds in both the HS and NT groups, accounting for 1.92% and 4.46% of the total volatile compounds, respectively, imparting a lilac aroma [38].

Ketones, resulting from the degradation of polyunsaturated fatty acids and amino acids, are commonly linked with creamy and fruity flavors [39]. Despite their high threshold, ketones generally play a coordinating role in the overall volatile flavor of meat products, positively influencing the volatile flavor profile of oyster *C. ariakensis*. Notably, three and six ketones were identified in the HS group, while six were found in the NT group. The relative content of ketones was higher in the NT group, which accounted for 24.09%, compared to 3.37% in the HS group. 

Esters, synthesized through esterification reactions involving acids derived from lipid or protein breakdown and alcohols, or through ester exchange reactions between fatty acids and ethanol in triglycerides, are known for their low thresholds and characteristic fruity and floral flavors. They play a significant role in contributing to the meat flavor profiles of aquatic products [40,41]. The reduced ester content in the HS group, compared to the NT group, indicated that heat stress had a detrimental impact on the flavor of oyster *C. ariakensis*, possibly due to the breakdown of heat-sensitive esters caused by heat stress.

Hydrocarbons are naturally produced through the enzymatic or non-enzymatic oxidation processes of long-chain fatty acids [39]. However, their contribution to overall flavor characteristics is limited due to their high odor thresholds [42]. Although the characteristic odor of hydrocarbons is not prominent, they play a crucial role in maintaining and coordinating aromas. Furthermore, unsaturated hydrocarbons can be converted into alcohols, aldehydes and ketones, thus impacting overall flavor [43]. In our study, the relative content of hydrocarbons and unsaturated hydrocarbons in the HS group (0.54%) was lower compared to the NT group (4.94%), indicating that heat stress adversely affects the flavor of oyster *C. ariakensis*.

Nitrogen-containing volatile flavor compounds are formed through the degradation of proteins, amino acids, and nucleic acids. The results demonstrated a higher relative content of nitrogen-containing compounds in the HS group (43.34%) compared to the NT group (25.25%). Pyridine, pyrrole, indole, pyrazole, and imidazole, which are nitrogen-containing volatile compounds, play a crucial role in enhancing overall flavor. Specifically, one pyridine (2-Aminopyridine), one pyrrole (Pyrrole, 2-methyl-5-phenyl-), one indole (1H-Pyrido [3,4-b] indole, 1-butyl-2,3,4,9-tetrahydro-) and one pyrazole (N-Nitroacetyl-3,5-dimethylpyrazole) volatile flavor compounds were identified in the HS group, while only one pyrazole (1H-Pyrazole, 4,5-dihydro-5-propyl-) and one pyrazole (1H-Benzimidazole, 3-oxide) were identified in the NT group. These findings suggested that heat stress may negatively impact the flavor characteristics of oyster *C. ariakensis* by stimulating the degradation of proteins, free amino acids, and nucleic acids. Notably, the reaction between amino acids and reducing sugars is one of the most important pathways for generating meat flavors, producing flavor compounds, such as furan, pyran, and pyrazine [44]. Compared to the NT group, HS had a lower relative content of furan and pyran. Specifically, two furans (2-Acetyl-2-methyltetrahydrofuran and 1-Propanone, 1-(2-furanyl)-) and one pyran (2H-Pyran, tetrahydro-2- [2- (methylene-cyclopropyl) ethoxy]-) were identified in the NT group, while only one furan (2-Acetyl-2-methyltetrahydrofuran) was identified in the HS group.

### 3.4. Correlation Analysis between Differential Metabolites and Sensory Properties

**Figure 6:**
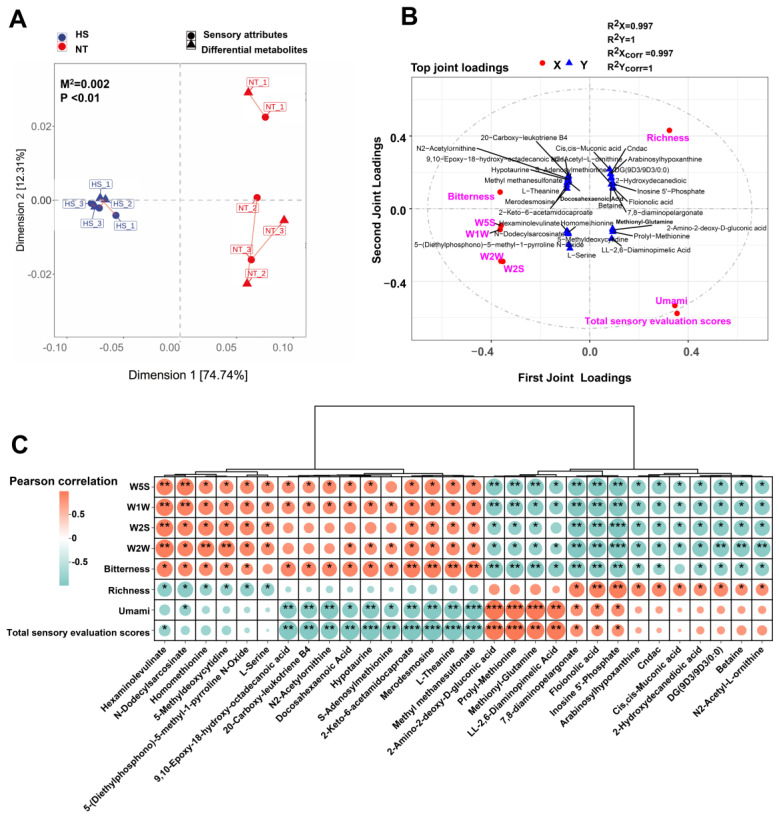
The correlation analysis between differential metabolites and sensory properties: (**A**) Procrustes analysis between differential metabolites and sensory properties; (**B**) the association between differential sensory properties and differential metabolites was evaluated by O2PLS; (**C**) Pearson’s correlation analysis for key differential metabolites and sensory properties. * *p* < 0.05, ** *p* < 0.01, *** *p* < 0.001. Sensory attributes include W5S, W1W, W2S, W2W, bitterness, richness, umami, and sensory evaluation. NT, normal group. HS, heat stress group.

Sensory properties depend on odor and taste precursor metabolites. To explore the relationship between sensory properties and these precursors, correlation analysis was conducted using differential responses from E-tongue E-nose, sensory evaluation scores, along with differential precursors from LC–MS. Procrustes Analysis was first performed to assess the consistency and correlation between differential E-tongue, E-nose responses, sensory evaluation scores and precursors. As shown in Figure 6A, significant associations were found between sensory properties and precursors (m^2^ = 0.002, *p* < 0.01). To identify key precursors contributing to sensory properties of oysters, O2PLS model analysis on differential E-tongue, E-nose responses, sensory evaluation scores, and precursors was conducted. O2PLS (two-way orthogonal partial least squares (O2PLS) analysis yielded R^2^X, R^2^Y, R^2^Xcorr and R^2^Ycorr values of 0.997, 1, 0.997 and 1, respectively, indicating the suitability of the model for further analysis (Figure 6B). Based on the O2PLS model results, we identified the top 30 precursors that exhibited a strong association with sensory properties of oysters. Subsequently, the correlation of the 30 precursors with sensory attributes was analyzed based on Pearson’s correlation analysis. Based on the criterion of *p* < 0.05, the results of Pearson’s correlation analysis revealed that inosine 5′-phosphate, floionolic acid, and 7,8-diaminopelargonate were positively correlated with richness, umami, and total sensory evaluation scores, but negatively correlated with W5S, W1W, W2S, W2W and bitterness. Additionally, precursors positively correlated with the total sensory evaluation scores included prolyl-methionine, methionyl-glutamine, 2-amino-2-deoxy-d-gluconic acid, and LL-2,6-diaminopimelic acid (Figure 6C). These results suggested that these precursors may be key metabolites in the changes in sensory attributes of oysters induced by heat stress. 

### 3.5. The Effect of Heat Stress on Metabolic Pathways in Oyster C. ariakensis

**Figure 7:**
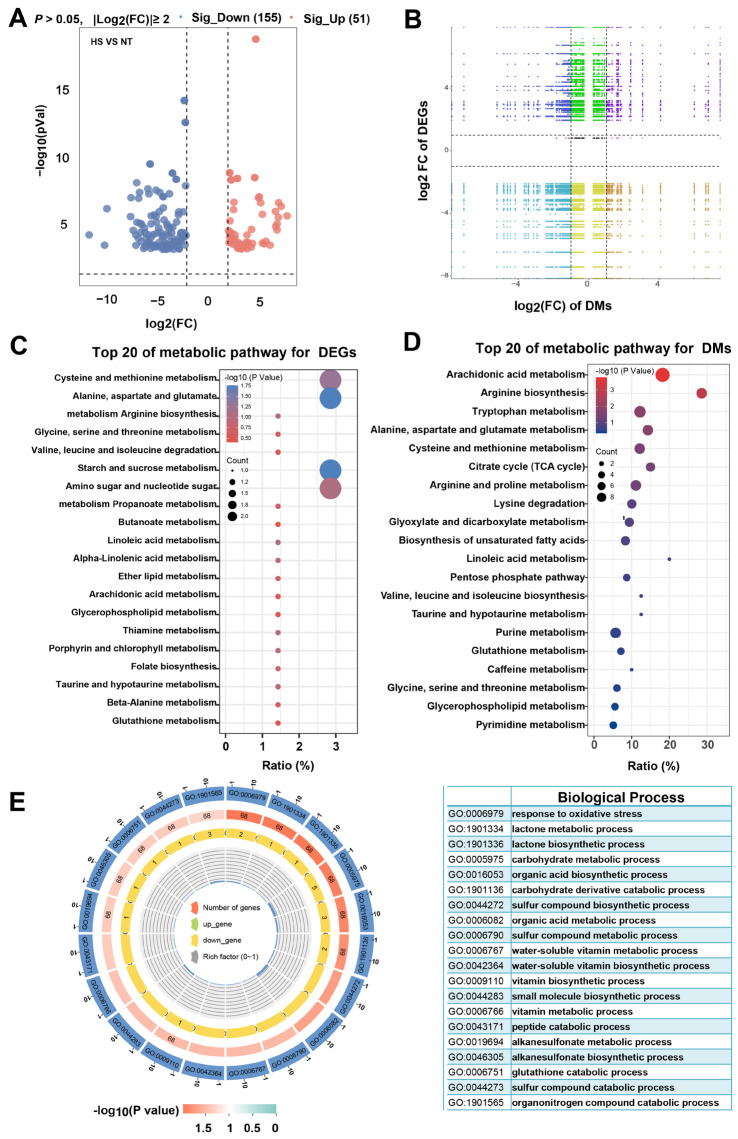
The effect of heat stress on metabolic pathways in oyster *C. ariakensis*: (**A**) Volcano plot; (**B**) Nine-quadrant diagram of DEGs and DMs; (**C**) Top 20 in KEGG enrichment analysis for DEGs; (**D**) Top 20 in KEGG enrichment analysis for DMS; (**E**) GO annotation for DEGs. NT, normal group. HS, heat stress group.

The combination of transcriptomics and metabolomics has emerged as a powerful tool for analyzing the underlying metabolic mechanisms of sensory properties’ formation, enabling comprehensive analysis of gene expression patterns and regulatory networks [45]. To investigate the potential metabolic mechanism of heat stress reduction in sensory properties of oyster, transcriptomic analyses were conducted on oyster from two groups. Compared to the NT group, the analysis revealed 166 differentially expressed genes (DEGs) identified under the threshold of |log_2_(FC)| > 2 and *p* < 0.05, among which 115 and 51 were down- and up-regulated genes in the HS group, respectively (Figure 7A and Table S4). A nine-quadrant diagram showed the association of DEGs with differential metabolites (DMs), where DEGs and DMs exhibiting a positive correlation in quadrants 3 and 7, while those showing a negative correlation are located in quadrants 1 and 9 (Figure 7B). Upon performing KEGG enrichment analysis for DEGs and DMs, shared same metabolic pathways were identified (Figure 7C,D). These pathways primarily encompassed glucose, lipid, and amino acid metabolism. The results of GO analysis for the DEGs indicated their significant enrichment in biological processes associated with glucose, amino acid, fatty acid, and organic acid metabolism. (Figure 7E). In addition, oxidative stress-related processes were also enriched. Therefore, the above results suggested that heat stress-induced flavor and taste reduction in oyster *C. ariakensis* were mainly related to glucose, lipid, amino acid organic acid metabolism and oxidative stress.

### 3.6. The Analysis of Key Genes Affecting Sensory Properties

**Figure 8:**
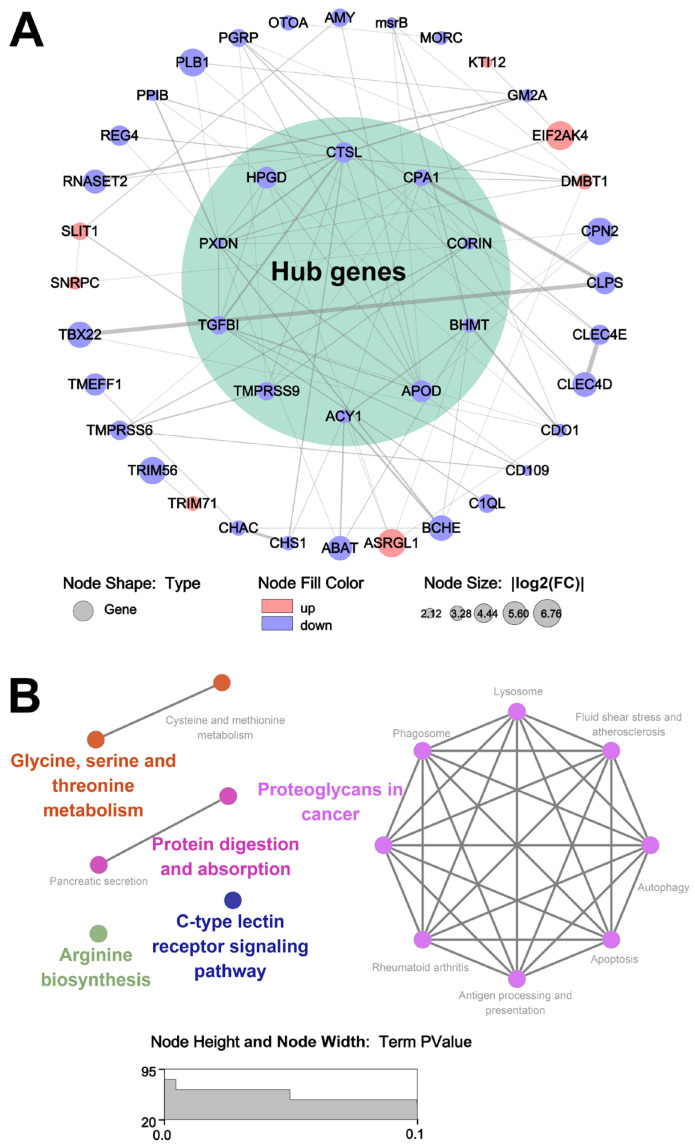
Protein–protein interaction: (**A**) Protein–protein interaction for DEGs; (**B**) KEGG enrichment analysis for hub genes.

To investigate the potential mechanisms of heat stress-induced sensory properties reduction in oysters, a protein–protein interaction (PPI) network was constructed. The ten hub genes (*CTSL*, *CPA1*, *CORIN*, *BHMT*, *APOD*, *ACY1*, *TMPRSS9*, *TGFBI*, *PXDN*, and *HPGD*) were selected from the PPI network by using the cytoHubba plug-ins (Figure 8A). The expression levels of the hub genes were significantly decreased in the HS group compared to the NT group. KEGG enrichment analysis showed that the hub genes were mainly enriched in organic acid metabolism and oxidation-related cellular processes (Figure 8B).

The redox state is crucial for maintaining normal cellular metabolism. The peroxidasin (PXDN) is a peroxidase that catalyzes the reduction of H_2_O_2_ to H_2_O, thereby reducing intracellular ROS levels [46]. The protein encoded by methionine sulfoxide reductase (msrB) has methionine residues on its surface that are readily oxidized by ROS, thereby mitigating ROS damage [47]. Additionally, the lack of *msrB* exacerbates GSH depletion and lipid peroxidation, leading to severe oxidative damage. In this study, compared to the NT group, *PXDN* and *msrB* were down-regulated, and increased ROS levels were observed in the HS group (Figure 2B), indicating that heat stress induced oxidative stress in oyster *C. ariakensis*.

Metabolic state in vivo plays a crucial role in the formation of sensory properties in meat. The amino-acylase 1 (*ACY1*) is a cytosolic, homo-dimeric, zinc-binding enzyme that catalyzes the hydrolysis of N-acyl-L-amino acids to L-amino acids and acyl group for the catabolism and salvage of acylated amino acids [48]. The betaine homocysteine methyltransferase (*BHMT*) catalyzes the formation of the amino acid methionine from homocysteine using the choline metabolite betaine as the methyl donor [49]. The cysteine dioxygenase 1 (*CDO1*) can catalyze l-cysteine to produce cysteine sulfinic acid [50]. Cysteine sulfinic acid is further decomposed into taurine and sulfate, which alleviates cysteine accumulation. Additionally, both *BHMT* [49] and *CDO1* [50] are involved in regulating lipid metabolism. In this study, compared to the NT group, the expression of *ACY1*, *BHMT*, and *CDO1* was down-regulated, indicating that heat stress may have caused metabolic disturbances in oyster *C. ariakensis*.

### 3.7. Proposed Formation Pathways for Sensory Properties

**Figure 9:**
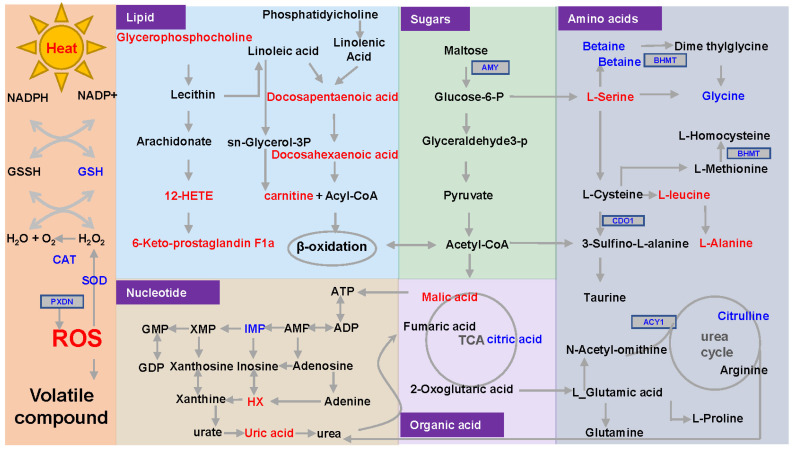
Underlying sensory properties formation network. Red font indicates up-regulating gene or metabolite, blue font indicates down-regulating gene or metabolite. ROS, Reactive Oxygen Species; SOD, superoxide dismutase; CAT, catalase; PLB1, phospholipase B1; AMY, amylase; BHMT, betaine homocysteine methyltransferase; CDO1, cysteine dioxygenase 1; ACY1, amino-acylase 1. PXDN, peroxidasin.

A metabolic network associated with sensory properties was constructed, which consisted of five components involving lipid, glucose, amino acid, organic acid, nucleotide, and antioxidant metabolism networks (Figure 9). The formation of sensory properties is intricately linked to metabolic processes [5,7]. Several studies have shown that heat stress induces metabolic abnormalities and increases the consumption of ATP [13,14], which is provided by the breakdown of lipids, glucose, and proteins and metabolized by the TCA cycle. ADP generated from ATP hydrolysis is metabolized to produce bitter Hx, which explains why heat stress caused a decrease in IMP as well as an increase in bitter Hx in oyster *C. ariakensis*. In addition, protein degradation can generate bitter amino acids and peptides, which is also a factor in the increase of bitter flavor under heat stress. Secondly, heat stress decreased the activities of antioxidant enzymes (SOD and CAT), down-regulated the expression level of antioxidant genes (*PXDN* and *msrB*) and increased the content of ROS in oyster *C. ariakensis*, indicating the occurrence of oxidative stress. In this state of oxidative stress, lipids, especially unsaturated fatty acids, are readily oxidized to produce compounds, such as aldehydes, ketones, and alcohols, which are believed to play an important role in odor formation [10]. The results in the study show that, under heat stress conditions, the unsaturated fatty acid content in oyster *C. ariakensis* was reduced, and unpleasant odor compounds, such as isopropanol, were generated. In short, heat stress negatively affected the sensory properties of oyster *C. ariakensis*, which may be due to heat stress-induced metabolic abnormalities, production of oxidative stress, and accumulation of bitter substances and unpleasant odor compounds. This study provides insights into understanding the mechanism of heat stress on oyster sensory properties’ reduction.

## 4. Conclusions

In the study, chronic heat stress reduced the sensory properties of oysters. The metabolic network constructed based on differential metabolites and differentially expressed genes showed that the negative effects of chronic heat stress on the sensory properties of oysters were related to oxidative stress, protein degradation, lipid oxidation and nucleotide metabolism. These findings offer valuable insights into the effects of heat stress on the sensory properties of oysters.

## Data Availability

The original contributions presented in the study are included in the article/supplementary material, further inquiries can be directed to the corresponding author.

## Supplementary Materials

The following supporting information can be downloaded at: https://www.mdpi.com/article/10.3390/foods13132004/s1, Table S1: The scoring standard of sensory evaluation; Table S2: The differential metabolites between HS and NT group; Table S3: The profiles of the volatile flavor compounds in HS and NT groups; Table S4: The differentially expressed genes between HS and NT groups.
